# Impact of social deprivation on cancer occurrence and cardiovascular events in systemic sclerosis

**DOI:** 10.1093/rap/rkag094

**Published:** 2026-08-04

**Authors:** Amy Turnbull, Ula Tymoszuk, Fiona A Pearce, Benjamin G Faber, Shaney L Barratt, Sue Farrington, Nick Jeffries-Owen, Ami A Shah, Clare Pain, Christopher P Denton, John D Pauling

**Affiliations:** Bristol Medical School, University of Bristol, Bristol, UK; Department of Internal Medicine, Bristol Royal Infirmary, University Hospitals Bristol and Weston NHS Foundation Trust, Bristol, UK; Scleroderma and Raynaud’s UK, London, UK; Lifespan and Population Health, School of Medicine, University of Nottingham, Nottingham, UK; Department of Rheumatology, North Bristol NHS Trust, Bristol, UK; Musculoskeletal Research Unit, Translational Health Sciences, Bristol Medical School, University of Bristol, Bristol, UK; MRC Integrative Epidemiology Unit, University of Bristol, Bristol, UK; Department of Respiratory Medicine, North Bristol NHS Trust, Bristol, UK; Scleroderma and Raynaud’s UK, London, UK; Scleroderma and Raynaud’s UK, London, UK; Division of Rheumatology, Johns Hopkins University School of Medicine, Baltimore, MD, USA; Department of Paediatric Rheumatology, Alder Hey Children’s NHS Foundation Trust, Liverpool, UK; Centre for Rheumatology and Connective Tissue Diseases, Division of Medicine, University College London, London, UK; Department of Rheumatology, North Bristol NHS Trust, Bristol, UK; Musculoskeletal Research Unit, Translational Health Sciences, Bristol Medical School, University of Bristol, Bristol, UK

**Keywords:** social deprivation, systemic sclerosis, cancer, cardiovascular, comorbidities

## Abstract

**Objectives:**

Cardiovascular events (CVEs) and malignancy are important non-disease-related causes of mortality in SSc. We evaluated secondary care utilisation and impact of deprivation on comorbidity patterns in SSc in England.

**Methods:**

We analysed secondary care service utilisation in SSc using Hospital Episode Statistics for England for 2024. Cases were linked to Index of Multiple Deprivation deciles. Myocardial infarction (MI), lung cancer, breast cancer, melanoma rates and short-term all-cause mortality were explored, alongside comparisons with SLE and RA.

**Results:**

In 2024, ≈6000 people with SSc (84.6% female, 70.8% between 51 and 80 years of age, 73.3% White ethnicity) accessed secondary healthcare in England. Older SSc patients were overrepresented in less-deprived postcodes (*P* < 0.001). MI, lung cancer, breast cancer and melanoma were more common in SSc compared with unmatched estimates in the general population. Lung cancer was more frequent among less-deprived patients (*P* < 0.001). The all-cause mortality attrition rate was ≈3% in SSc over 3 months (30.8% with lung cancer and >60% with melanoma). Rates of breast cancer were higher in SSc compared with SLE (*P* = 0.014) and RA (*P* = 0.012). There was a higher rate of lung cancer in SSc compared with SLE (*P* < 0.001). Melanoma rates were similar across diseases (≈0.2%).

**Conclusion:**

Secondary care utilisation in SSc suggests less deprived SSc patients are older. All-cause mortality is higher in SSc patients receiving cancer care. Cancer occurrence is higher in SSc compared with SLE and RA. Our findings might support healthcare services planning and cancer screening for SSc in England.

Key messages

- There are ≈6000 people utilizing secondary care services for SSc in the UK.
- Most patients with SSc are >50 years of age and live in less-deprived postcodes.
- SSc patients have higher cancer and myocardial infarction rates than non-SSc patients.

## Introduction

SSc is a rare systemic autoimmune rheumatic disease (SARD) [1, 2]. SSc has the highest disease-related mortality of the SARDs [3]. Although outcomes have improved, cardiovascular events (CVEs) and malignancy remain important causes of mortality [4, 5].

Socio-economic inequalities, often measured by the Index of Multiple Deprivation (IMD), have been established across multiple chronic diseases, including cardiovascular disease and cancer [6–8]. The IMD provides a composite measure of socio-economic status across domains including income, employment, education and health. A socio-economic gradient has been demonstrated across other SARDs, including RA and SLE [9]. Countries with a higher sociodemographic development level have a disproportionately higher burden of SARDs [10]. There is limited evidence on the impact of socio-economic status on SSc-related comorbidities.

Previous UK-wide population studies have used large, linked databases such as the Clinical Practice Research Datalink (CPRD) to investigate outcomes in SSc, including increased risks of lung cancer and coronary artery disease [11–13]. In contrast, fewer population-based studies have been conducted based on secondary healthcare records. Hospital Episode Statistics (HES) provide a comprehensive record of secondary care activity across England, enabling large-scale epidemiological studies to examine comorbidity patterns. However, to our knowledge, HES have not been used to assess socio-economic inequalities and comorbidity risk in SSc. Identification of the impact of inequalities in this cohort across England may help inform future strategies to plan services to abrogate them, improving patient care.

Against this backdrop, we aimed to examine the distribution of selected comorbidities in patients with SSc across England using secondary healthcare data and to explore whether socio-economic deprivation is associated with the risk of adverse outcomes.

## Methods

### Data source

We conducted a retrospective observational study using HES, accessed via NHS DigiTrials [14]. This database records all admissions, outpatient visits and emergency attendances across National Health Service (NHS) hospitals in England, updated monthly. Within NHS DigiTrials, data from all three HES datasets were combined, thus analyses included records from all three sources. All searches were conducted between August and December 2025 and results reflect the live HES dataset available at that time. HES is continuously updated, therefore serial data extracts yield slightly different patient counts (e.g. typically with attrition of patients following death). All data were de-identified, with any patient count <8 suppressed, values ≥8 rounded to the nearest five and output tables with patient counts <11 not displayed. Rounding errors account for slight discrepancies in patient counts in different search results and further analysis (with figures often rounded to the nearest five integer).

### Study population

Patients were identified using International Classification of Diseases, Tenth Revision (ICD-10) codes. All patients with a diagnostic code for SSc (M34) between 1 January and 31 December 2024 were identified from the HES. Comorbidities were selected based on expected need for secondary care services and prior UK-based epidemiological studies using CPRD [12, 13]. This included myocardial infarction (I21), lung cancer (C34), breast cancer (C50) and melanoma (C43). Additional data were exported for RA (M05) and SLE (M32). Data exported for these conditions were compared with the SSc patient count performed concurrently, with lower values providing insights into the gradual mortality-related attrition rate in SSc. The validity of routinely collected diagnostic codes for SSc has been previously evaluated, with a positive predictive value of the ICD-10 code M34 for SSc of 86.8% (95% CI 71.9, 95.6) [15].

Overlapping diagnoses of SSc and each comorbidity were manually calculated. Patients were classified as having both conditions if they had ≤1 episode coded with SSc and ≤1 with the comorbidity within their linked HES records, irrespective of whether these occurred in the same or separate admissions. The number of patients with both conditions was derived by manually subtracting patients with SSc alone and the comorbidity alone from combined totals. Population denominators were obtained from UK mid-year 2024 England population estimates published by the Office for National Statistics (ONS) [16].

A repeat analysis was undertaken after 3 months, to further evaluate gender differences. As the database is dynamic, patients who are deceased are removed from the dataset, leading to a decrease in the total number of patients identified within the same timeframe. However, this provided insights into the estimated attrition rates related to all-cause mortality. Counts used for comparison are highlighted in the relevant results.

### Statistical analysis

Patient demographics (age, sex, ethnicity and region) and comorbidity counts were extracted from the HES and analysed descriptively. Associations between deprivation and disease burden were assessed using Spearman’s rank correlation. Results therefore represent population-level associations and cannot be interpreted at an individual level. Categorical variables were compared using chi-squared tests, or Fisher’s exact tests where expected cell counts were <5. IMD deciles were dichotomised for some analyses due to low patient counts. Age was analysed in two strata to reflect older age: <70 years and ≥70 years. In the general population dataset, age categories available were ≤69 years and 70 years. These were treated as equivalent to the strata used in the SSc cohort; any discrepancy is limited to the boundary year and is unlikely to significantly affect results in this study. Approximate mean ages were estimated from group age-band frequencies using category midpoints.

Residential postcodes were linked to the 2019 IMD, a composite measure of socio-economic status across deciles, with lower IMD deciles representing greater deprivation. For analysis of comorbidities, IMD deciles were dichotomised into more deprived (IMD 1–5) and less deprived (IMD 6–10), due to small patient counts for SSc, as expected for a designated orphan disease. Statistical analyses were performed in SPSS Statistics version 30.0 (IBM, Armonk, NY, USA).

### Ethics and data governance

Data access was obtained under a data sharing agreement with NHS Digital’s service. All data were anonymised and no patient identifiers were available to the research team. Ethical approval and patient consent were not required for this study of routinely collected, fully de-identified secondary care NHS data provided as part of an existing license agreement with NHS DigiTrials.

## Results

### Study demographics

**Figure 1:**
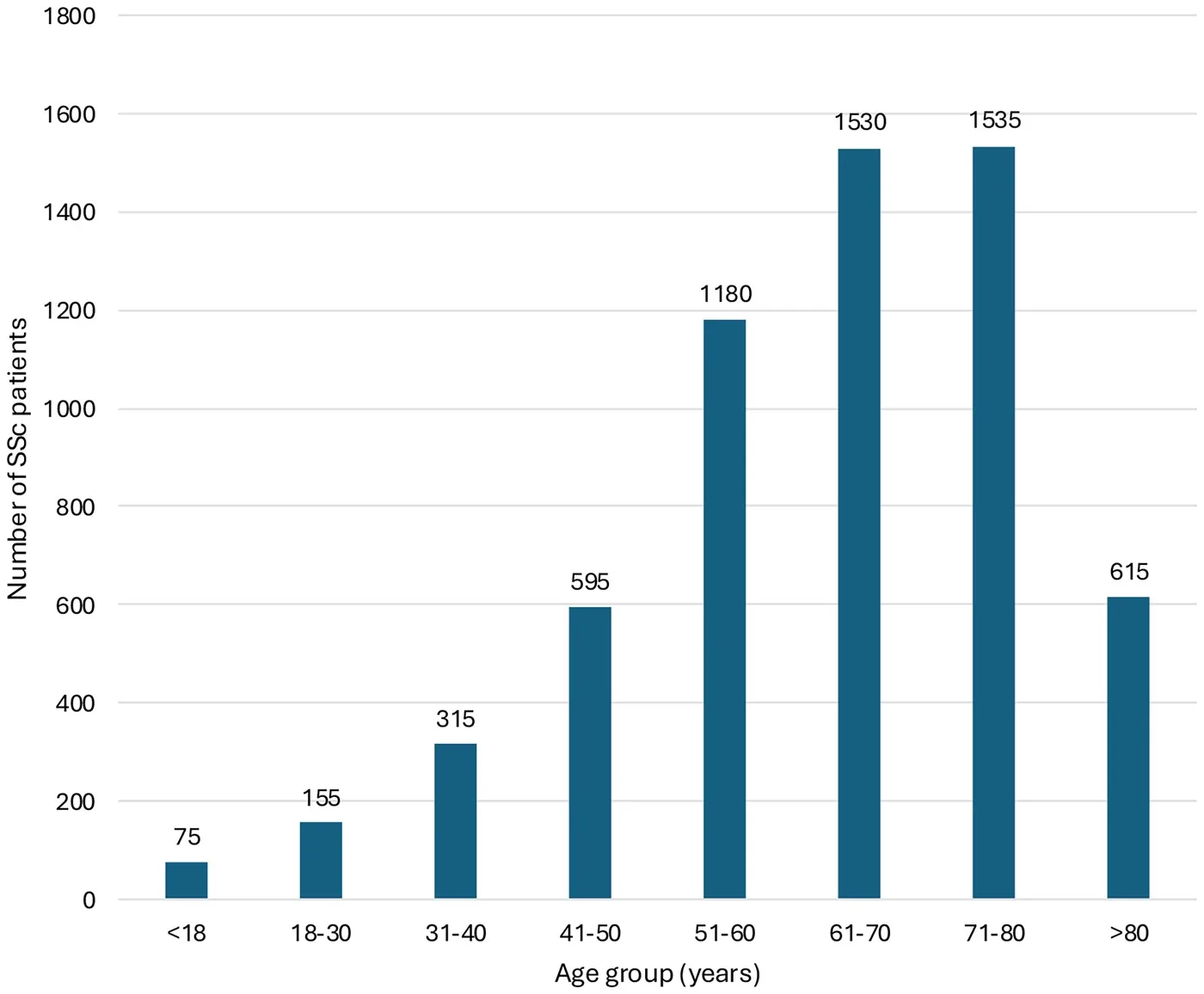
Age distribution of patients with SSc who accessed secondary care in 2024. Bars represent the total number of SSc patients within each age group, with counts shown above each bar

Our initial searches revealed 6005 people with a diagnosis of SSc as having accessed secondary healthcare in England (1 January 2024–31 December 2024) from a total of 24 777 205 individuals utilising secondary care services. Consistent with an expected male:female ratio of 1:5.5, our cohort was comprised of 925 men (15.4%) and 5080 women (84.6%). The male:female distribution was consistent across all regions. The age distribution was skewed towards older adults, with the majority being ages 51–80 years (70.8%), including 25.5% ages 61–70 years and 25.6% ages 71–80 years (Fig. 1). The proportion of SSc patients >80 years of age was 10.3%. There were relatively fewer cases in younger individuals, with 156 (2.6%) ages 18–30 years and 78 (1.3%) < 18 years. This distribution was consistent across all regions. Consistent with regional population sizes, London (15.8%), the South East (14.8%) and the North West (14.4%) contributed the largest proportions of patients, with the North East accounting for only 4.0%.

Most SSc patients in this cohort were White (82.3%). Among non-White patients, Asian/Asian British represented the most reported ethnicity (9.3% of patients), more than double the number of patients reporting Black ethnicity. Ethnicity data were missing or unreported in ≈10% of people with SSc.

### Socio-economic gradient

**Figure 2:**
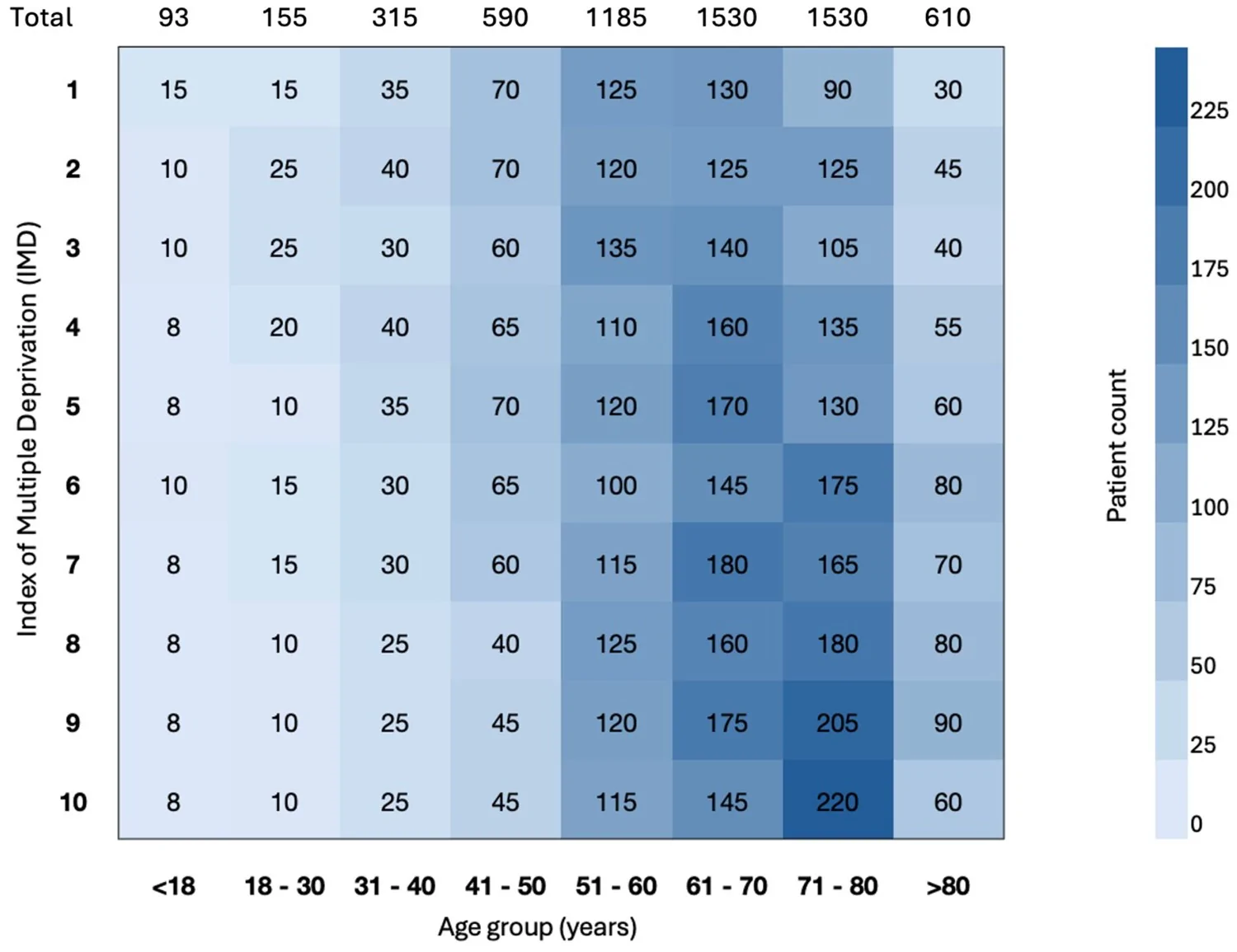
Heat map showing the distribution of patient counts across age groups and IMD deciles. Columns represent age groups (in years) and rows represent IMD deciles (1 = most deprived, 10 = least deprived). Cell shading corresponds to the number of patients in each age–deprivation stratum, with darker shading indicating higher patient counts. Values within cells indicate the absolute patient numbers and column totals are shown above the heat map

There was a strong positive correlation between SSc and level of deprivation (ρ = +0.91, *P* < 0.001), with higher counts observed in less-deprived IMD deciles (Table 1). There was strong evidence of an association between age and deprivation (χ^2^  *P* < 0.001). Older patients were overrepresented in less-deprived groups [>80 years: IMD 1 (−3.0), IMD 10 (+4.8)]. In the broader secondary care England population, there was also evidence of an association between age group and IMD decile, with older age groups more likely to reside in less-deprived areas. However, the association was stronger in SSc (Cramer’s V = 0.071, Spearman’s ρ = 0.153) than the overall secondary care population (Cramer’s V = 0.005, Spearman’s ρ = 0.133), suggesting a stronger relationship between older age and higher IMD decile within the SSc cohort. There was a clear age-deprivation gradient in SSc (Fig. 2), with younger patients (<50 years) overrepresented in more-deprived areas and older patients (>50 years) disproportionately represented in less-deprived areas. For example, patients >80 years of age were significantly underrepresented in IMD 1 (standardised residual −3.0) but overrepresented in IMD 10 (+4.8). Mantel–Haenszel testing provided strong evidence of a linear trend (*P* < 0.001). The proportion of younger patients decreased progressively with increasing deprivation, while the proportion of older patients was higher in less-deprived areas (Fig. 2).

**Table 1:** Comparison of adverse outcomes in patients with SSc *vs* the non-SSc population, stratified by age (<70 and ≥70 years).

Outcome	SSc <70 years, *n*/*N* (%)	Non-SSc <70 years, *n*/*N* (%)	*P*-value	SSc ≥70 years, *n*/*N* (%)	Non-SSc ≥70 years, *n*/*N* (%)	*P*-value
Lung cancer	25/3850 (0.65%)	14 980/50 586 801 (0.03%)	<0.001	25/2150 (1.16%)	20 940/8 027 300 (0.26%)	<0.001
Melanoma	0/3850 (0.00%)	13 190/50 586 801 (0.03%)	0.632	10/2150 (0.47%)	12 435/8 027 300 (0.15%)	0.002
Breast cancer	53/3850 (1.38%)	67 720/50 586 801 (0.13%)	<0.001	25/2150 (1.16%)	31 615/8 027 300 (0.39%)	<0.001
Myocardial infarction	25/3850 (0.65%)	43 805/50 586 801 (0.09%)	<0.001	35/2150 (1.63%)	39 135/8 027 300 (0.49%)	<0.001

N represents the total number of individuals in each cohort and age stratum. *P*-values compare the proportion of each outcome between SSc and non-SSc groups within each age stratum (chi-squared test or Fisher’s exact test where appropriate).

### Cancer occurrence in SSc

Among patients with SSc, 65/6005 (1.08%) patients accessed secondary care services for lung cancer compared with 32 920 (0.06%) in the non-SSc population (χ^2^  *P* < 0.001), with a relative risk of 19.27 (95% CI 15.13, 24.55). The difference between the SSc and non-SSc population was significant in patients both greater than and less than age 70 years (Table 1). A repeat analysis 3 months later was performed that identified 5825 patients with SSc, suggesting an overall all-cause mortality attrition rate among SSc patients utilising secondary care services of ≈3%. The number of SSc patients with lung cancer decreased to 45/5825 (0.77%) over this period, indicating an all-cause mortality attrition rate of 30.8% for SSc patients with lung cancer over 3 months.

Breast cancer was identified in 90/6005 patients with SSc compared with 99 330/58 614 096 without SSc (1.5% *vs* 0.17%; *P* < 0.001), although the relative risk of breast cancer could not be determined as biological sex was not captured on the initial search. The attrition rate at 3 months was 15/90 (16.7% of the total number of SSc patients receiving breast cancer care). At this 3-month data acquisition, there were 75/5825 (1.29%) SSc patients identified as utilising secondary care services for breast cancer (100% female). Comparing only female patients, breast cancer was significantly more common in those with SSc than in those without SSc (1.63% *vs* 0.35%; *P* < 0.001). While some of this discrepancy likely relates to the older age of our SSc population compared with the general population utilising secondary care as a whole, a higher rate of breast cancer was still identified in SSc when comparing patients less than or greater than age 70 years (Table 1).

Melanoma occurred in 25/6005 patients with SSc compared with 25 610/58 614 096 without SSc (0.42% *vs* 0.04%; χ^2^  *P* < 0.001), with a relative risk of 9.53 (95% CI 6.44, 14.09). All melanoma cases occurred in patients >70 years of age. Consistent with known poorer outcomes in melanoma, our 3-month analysis revealed an attrition rate of 60%, with only 10/5825 (0.17%) patients with SSc and melanoma in the later analysis (100% female).

### Cardiovascular disease in SSc

Myocardial infarction (MI) was recorded in 65/6005 (1.08%) SSc patients and 82 935 (0.14%) of the non-SSc population. The relative risk of MI was 7.65 (95% CI 6.01, 9.74). While this group data might be partly explained by our SSc population being older than the general population utilising secondary care services as a whole, the rates of MI were higher in SSc for those less than and greater than 70 years of age (Table 1). In our 3-month analysis, 55/5825 (0.9%) patients with SSc were identified with MI and 81.8% were female. This represents an attrition of 15.4% (10/65), following all-cause mortality. In our 3-month analysis that permitted subanalysis according to biological sex, MI was significantly more common in men with SSc than in the general population [10/905 (1.10%) *vs* 53 510/28 723 434 (0.19%); Fisher’s exact test *P* < 0.001]. MI was also more common in women with SSc [45/4920 (0.91%) *vs* 26 165/29 890 842 (0.09%); Fisher’s exact test *P* < 0.001]. These risk estimates were not adjusted for important confounders, such as age, due to limitations in the format of the dataset.

### Association between deprivation and comorbidity risk

IMD deciles were dichotomised to stratify by socio-economic status. Against our expectations, lung cancer in SSc was significantly more frequent in patients from less-deprived areas (1.6% *vs* 0.5%; *P* < 0.001). The distribution of lung cancer cases across IMD deciles differed between SSc and the overall secondary care population (Table 2). There was no strong evidence for differences between deprivation and breast cancer (*P* = 0.65). There was weak evidence of a trend toward higher rates of MI among SSc patients living in wealthier areas (IMD 6–10 *vs* 1–5) compared with those in more-deprived areas (1.6% *vs* 0.9%; *P* = 0.18). Melanoma rates were similar in SSc patients irrespective of lower or higher deprivation (0.47% *vs* 0.36%; *P* = 0.50).

**Table 2:** Distribution of SSc and comorbidities across IMD deciles in England (2024).

Characteristics	Total	IMD 1	IMD 2	IMD 3	IMD 4	IMD 5	IMD 6	IMD 7	IMD 8	IMD 9	IMD 10
Population that accessed secondary healthcare, *n* (%)	24 777 205	2 564 730 (10.4)	2 532 420 (10.2)	2 521 565 (10.2)	2 490 905 (10.1)	2 472 980 (10.0)	2 505 675 (10.1)	2 462 625 (9.9)	2 449 400 (9.9)	2 421 560 (9.8)	2 355 340 (9.5)
SSc, *n* (%)	6005	510 (8.5)	555 (9.2)	555 (9.3)	585 (9.7)	605 (10.1)	620 (10.3)	645 (10.7)	630 (10.5)	675 (11.2)	625 (10.4)
Lung cancer in SSc, *n* (%)	65	0 (0.0)	5 (7.7)	5 (7.7)	5 (7.7)	0 (0)	10 (15.4)	10 (15.4)	10 (15.4)	10 (15.4)	10 (15.4)
Lung cancer in the general population, *n* (%)	35 985	4885 (13.6)	3965 (11.0)	3790 (10.5)	3760 (10.4)	3620 (10.1)	3390 (9.4)	3385 (9.4)	3310 (9.2)	3130 (8.7)	2750 (7.6)
Melanoma in SSc, *n* (%)	25	0 (0.0)	5 (20.0)	5 (20.0)	0 (0.0)	0 (0.0)	5 (20.0)	5 (20.0)	0 (0.0)	0 (0.0)	5 (20.0)
Melanoma in the general population, *n* (%)	25 635	1205 (4.7)	1335 (5.2)	1710 (6.7)	2140 (8.3)	2525 (9.8)	2890 (11.3)	3100 (12.1)	3270 (12.8)	3575 (13.9)	3885 (15.2)
Breast cancer in SSc, *n* (%)	90	5 (5.6)	10 (11.1)	5 (5.6)	15 (16.7)	5 (5.6)	10 (11.1)	5 (5.6)	10 (11.1)	10 (11.1)	15 (16.7)
Breast cancer in the general population, *n* (%)	99 420	7245 (7.3)	7940 (8.0)	8870 (8.9)	9250 (9.3)	9910 (10.0)	10 660 (10.7)	10 915 (11.0)	11 235 (11.3)	11 590 (11.7)	11 805 (11.9)
MI in SSc, *n* (%)	65	0 (0)	5 (7.7)	10 (15.4)	10 (15.4)	10 (15.4)	10 (15.4)	5 (7.7)	5 (7.7)	10 (15.4)	10 (15.4)
MI in the general population, *n* (%)	83 000	9440 (11.4)	8740 (10.5)	8475 (10.2)	8610 (10.3)	8300 (10.0)	8450 (10.2)	8285 (10.0)	7840 (9.4)	7665 (9.2)	7195 (8.7)

Percentages represent proportions within each IMD decile relative to the total SSc cohort or the total population that accessed secondary healthcare (IMD 1 = most deprived, IMD 10 = least deprived).

### Comparison with other SARDs

**Figure 3:**
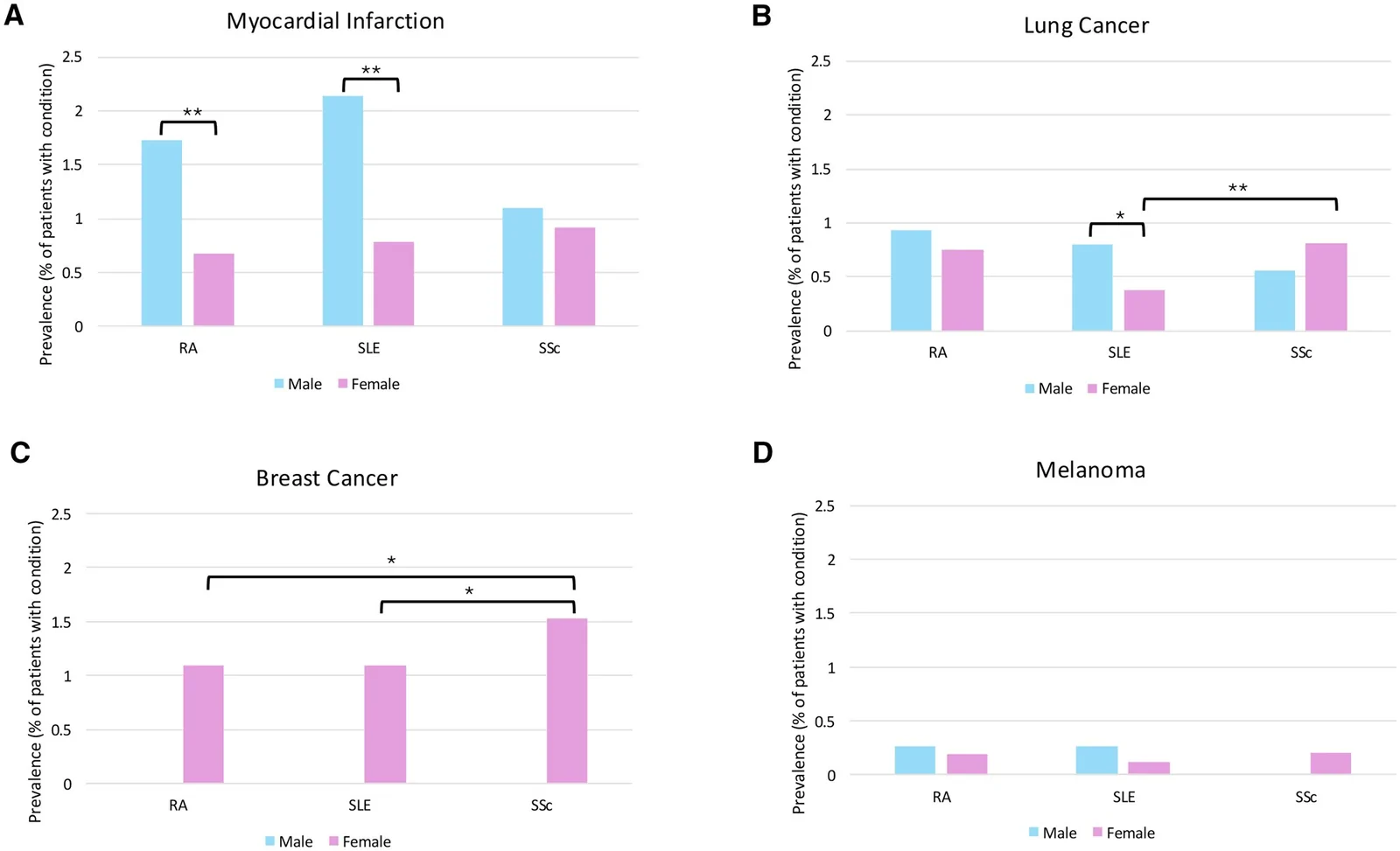
Prevalence of disease outcomes in SSc compared with RA and SLE in 2024. Sex-specific prevalence (%) of **(A)** MI, **(B)** lung cancer, **(C)** breast cancer (female patients only) and **(D)** melanoma across RA, SLE and SSc cohorts. Prevalence was calculated as the percentage of patients with each condition, separately for males and females, using sex-specific denominators. Male–female comparisons within each disease and between disease groups were performed using chi-squared tests (*P < 0.05, **P < 0.001)

At the time of our analysis, 28 045 patients with RA were identified as having accessed secondary care services in 2024. This was compared with 5825 patients with SSc identified from the same data extract (Fig. 3). A total of 17 920 patients with SLE were identified. The SLE cohort was younger overall, with an estimated mean age of 53.5 years (median age category 51–60 years), compared with 66.6 years in the SSc group. In contrast to SSc, Spearman’s rank correlation demonstrated a strong negative correlation between IMD decile and SLE cases (ρ = −0.86, *P* = 0.002), indicating an overall higher number of SLE cases in more-deprived deciles.

There was a similar trend for RA; however, this did not reach statistical significance (ρ = −0.11, *P* = 0.76). The estimated mean age for RA was 66.6 years (median age category 61–70 years).

Rates of MI in RA [270 (0.96%)] did not differ when compared with SSc [55 (0.94%), *P* = 0.90]. Similarly, rates of MI in SLE [160 (0.89%)] were not significantly different from that in SSc (*P* = 0.72). Likewise, lung cancer occurred in 225 (0.80%) patients with RA, compared with 45 (0.77%) patients with SSc, with no significant difference between groups (*P* = 0.818). In contrast, there was a significant difference in the proportion of patients with lung cancer between the SSc and SLE groups [45/5825 (0.80%) *vs* 45/17 920 (0.30%), respectively; *P* < 0.001].

When comparing women, there was a significantly higher prevalence of breast cancer in SSc compared with RA [75/4920 (1.52%) *vs* 225/20 535 (1.10%), respectively; *P* = 0.012]. There was also a significantly higher proportion of women with breast cancer with SSc compared with SLE [75/4920 (1.52%) *vs* 175/16 045 (1.09%), respectively; *P* = 0.014].

Overall melanoma rates were similar in RA [60 (0.21%)], SLE [30 (0.17%)] and SSc [10 (0.17%)] (*P* > 0.05).

## Discussion

We report a large observational cross-sectional study using HES, providing insights into healthcare utilisation and comorbidities associated with SSc at a population level in England. To our knowledge, this is the first study to utilise NHS DigiTrials to access HES data for England to explore SSc comorbidities. Using this platform, we undertook crude analyses of the relative burden of MI, lung cancer, melanoma and breast cancer in SSc, with comparisons against the general population and selected comparator SARDs (RA and SLE). Secondary analyses at 3 months provided insights into attrition due to all-cause mortality within the dataset. We had not fully appreciated this effect at the study outset. Indeed, our initial analyses in August 2025 did not take into account the attrition that occurred due to all-cause mortality between January and August, which resulted in unanticipated reductions in each of our estimates across SSc, SLE, RA and the general population. Building search strategies in NHS DigiTrials was somewhat limited, although this proved valuable for undertaking preliminary hypothesis-generating analyses. NHS DigiTrials also has the advantage of providing insightful IMD and geographic data.

Our findings provide useful data on secondary care healthcare utilisation in SSc in England. The majority of patients receiving secondary care with SSc are older. SSc is rare in children (1.3% of the total SSc population). Meanwhile, the proportion of patients with SSc in each age category increases to a peak in the 70- to 80-year category, with 35.8% of the UK population of SSc patients being >70 years of age. This may impact on the ability of patients to attend secondary services, particularly if their care is coordinated at a distant specialist SSc centre. Our findings are consistent with previous work noting SSc to be 4.7 times higher in women, with the highest prevalence in the 70- to 84-year age group [17].

We identified an age–socio-economic gradient, with younger patients more likely to reside in more-deprived areas and older patients more likely to reside in less-deprived areas. This possibly reflects survival bias based on confounders associated with higher socio-economic status. HES data is unable to provide information on SSc subtypes or interventions. We observed a similar age–socio-economic gradient in the wider population, with older age groups slightly more represented in less-deprived deciles. Age and deprivation are not independent and may confound observed associations between deprivation and comorbidities in SSc. A retrospective study in England using ONS data that found the relationship between socio-economic deprivation and mortality differs by age in the general population, with the largest disparities in middle and older age groups [18]. An observational study of primary care records in England found that relative inequalities peak in middle age and change with age, indicating that socio-economic gradients manifest differently across different age groups [19]. These patterns may reflect differential survival and healthcare utilisation across different socio-economic groups, whereby individuals from areas of higher deprivation experience higher early mortality and are therefore underrepresented in older age groups.

International registries have described ethnic variation in disease incidence and outcomes but UK-specific data remain limited [20]. Other UK regional studies have found a similar proportion of White patients with SSc (83%) as our analysis but a higher proportion of South Asian patients [21]. Our findings should be interpreted with caution, as routinely collected healthcare records may be subject to incomplete recording and variation in coding. Diagnostic barriers among certain ethnic groups may also contribute.

Our findings regarding comorbidities are consistent with previous national cohort studies demonstrating an increased cardiovascular and malignancy burden in SSc [11, 12, 22]. For each comorbidity studied, the proportion of affected individuals was higher in the SSc cohort than in the non-SSc population (generally consistent for those less than and greater than 70 years of age). In 2024, ≈3% of patients with SSc in England were utilising secondary care services for either lung cancer, breast cancer or melanoma. The increased prevalence of MI in SSc may reflect the combined effects of chronic systemic inflammation, endothelial dysfunction and microvasculopathy, which are recognised features of SSc and may contribute to accelerated atherosclerosis and cardiovascular disease [2, 3, 13]. Similarly, the increased prevalence of lung cancer is biologically plausible given the recognised association between SSc-related fibrosis, chronic inflammation and malignancy [2, 4, 12]. The mechanisms underlying the increased prevalence of breast cancer and melanoma are less clearly established but may involve immune dysregulation, altered immune surveillance and increased healthcare contact [2, 3, 12].

It was not possible to adjust for confounders such as age in our analysis, and our reported relative risks must be interpreted with caution given our SSc population was older. The small number of events within the SSc cohort also resulted in wide CIs. Surveillance biases inherent in routinely collected secondary care data may also exist. Patients with SSc have more frequent healthcare contact, investigations and cross-sectional imaging, which may increase incidental detection of cancers compared with the general population, and there may be differences in screening uptake. Selected malignancies were chosen based on prior epidemiological studies and relevance to secondary care utilisation, however others, including haematological malignancies, were not assessed and may warrant further investigation. Nonetheless, our findings do broadly replicate the findings of earlier studies and highlight value in using NHS DigiTrials for hypothesis-generating research.

To our knowledge this is the first national study to examine the association between deprivation and comorbidities in SSc using secondary care data from England. Our study also provides comparative insights into malignancy and cardiovascular disease burden across other autoimmune disease cohorts. Socio-economic gradients have been described in other autoimmune diseases, including RA and SLE [23], with greater deprivation associated with higher prevalence and poorer outcomes. In our analysis, the more frequently observed lung cancer in less-deprived areas may partly reflect survival bias (individuals in more-deprived areas experiencing earlier mortality from all causes prior to SSc diagnosis), with the remaining patients being at lower risk of developing complications as they have survived long enough to develop SSc owing to other factors. Differences in screening uptake may also contribute, as national screening analyses show that individuals from less-deprived quintiles are more likely to attend lung cancer screening [24]. Our findings may also reflect differences in risk factors for lung cancer in SSc compared with the general population. Environmental factors influence the prevalence and outcomes in autoimmune rheumatic diseases [23]. It may also reflect poorer availability of access to secondary care in areas of higher deprivation, limiting early diagnosis and coding. As we were unable to adjust for potential confounders, further population-level studies are needed to explore this association, which might have implications for cancer screening in SSc.

The key limitation of this study was the dynamic nature of the database, with removal of subjects from the database after death. Therefore, temporal analysis is not possible and the most accurate analysis provides only a snapshot in time. Furthermore, for rare diseases like SSc, low patient counts are hidden to ensure data is de-identified, which resulted in small rounding errors within the analyses. These limitations meant that additional searches on DigiTrials run at a different time resulted in different patient counts for the same time period. However, analyses have been performed on the relevant comparable counts depending on the time they were extracted. Furthermore, we were unable to examine adverse outcomes simultaneously stratified by age and IMD, limiting assessment of age–socio-economic gradients for individual outcomes. Differences in age distribution between disease cohorts (SSc, RA, SLE) are important to consider when comparing disease outcomes. Approximate age estimates suggest the SLE cohort was younger than the RA and SSc cohorts, which may partially reflect the lower observed cancer and MI prevalence. Future work using larger datasets would enable more granular analyses of adverse outcomes across combined age and deprivation groups. Finally, as with other population-based datasets, there may be diagnostic coding inaccuracies and discrepancies between regions, particularly in outpatient departments. However, previous validation work has demonstrated a high positive predictive value (86.8%) for the ICD-10 M34 coding for SSc, supporting its use in SSc case identification [15].

Our analysis has been useful for exploring secondary care utilisation in SSc and has provided insights into the association between deprivation and outcomes. Our analysis has supported hypothesis generation for further work exploring the impact of social deprivation on comorbidities in SSc, with valuable crude comparisons with both the general population and other SARDs. Our findings highlight the need for additional robustly designed studies examining the impact of socio-economic factors when planning SSc care, cancer screening programs and the development of targeted preventative strategies.

## Data Availability

The data underlying this article were accessed through the NHS DigiTrials service. Restrictions apply to the availability of these data, which were used under licence for this study and are not publicly available. Our raw data can be made available upon request.
